# ASGR1 is a promising target for lipid reduction in pigs with PON2 as its inhibitor

**DOI:** 10.1016/j.isci.2024.110288

**Published:** 2024-06-17

**Authors:** Yunjun Yin, Jun Liu, Jia Yu, Dingcai Dong, Fei Gao, Libao Yu, Xuguang Du, Sen Wu

**Affiliations:** 1 State Key Laboratory of Animal Biotech Breeding, College of Biological Sciences, China Agricultural University, Beijing 100193, China; 2 Sanya Institute of China Agricultural University, Sanya 572024, China; 3 The Eighth Medical Center of PLA General Hospital, Beijing 100094, China

**Keywords:** Human metabolism, Genetics

## Abstract

Although the role of asialoglycoprotein receptor 1 (ASGR1) in lowering lipid levels is well established, recent studies indicate that ASGR1 inhibition can cause unexpected liver damage in pigs, raising a serious issue about whether ASGR1 can be a good target for treating ASCVD. Here, we utilized the CRISPR-Cas9 system to regenerate ASGR1-knockout pigs, who displayed decreased lipid profiles without observable liver damage. This was confirmed by the lower levels of serum ALT and AST, reduced expression of inflammation markers, and normal histological morphology. Also, we implemented immunoprecipitation combined with mass spectrometry (IP-MS) and discovered that paraoxonase-2 (PON2) can interact with and significantly degrade ASGR1 in a dose-dependent manner. This degradation reduced lipid levels in mice, accompanied by little inflammation. Our study highlights the effectiveness and safety of degrading ASGR1 to reduce lipid levels in pigs and provides a potential inhibitor of ASGR1.

## Highlights

• ASGR1^+/−^ pigs present a low lipid profile with no obvious inflammation
• PON2 binds ASGR1 and induces its degradation
• Overexpression of PON2 decreases ASGR1 and blood cholesterol levels in mice

## Graphical abstract

**Figure:**
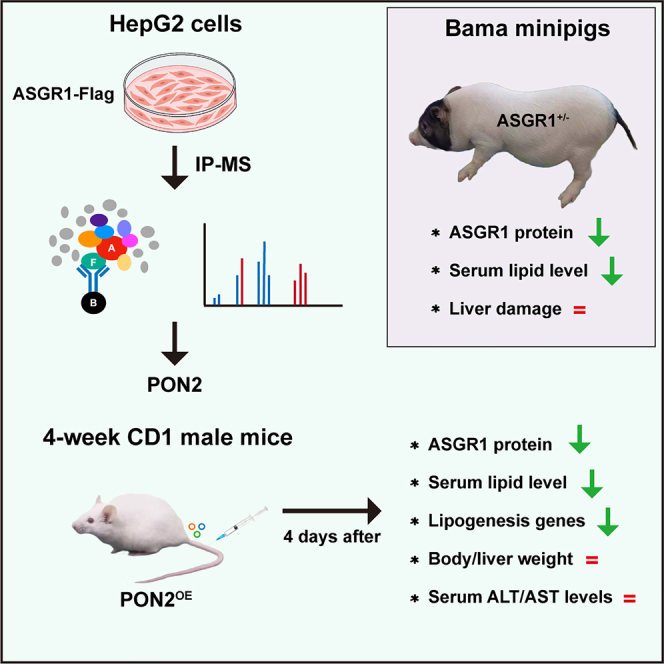

## Introduction

Atherosclerotic cardiovascular disease (ASCVD), the leading cause of morbidity and mortality globally, is mainly driven by elevated levels of non-high-density lipoprotein cholesterol (non-HDL-C) and inflammation.^1^^,^^2^^,^^3^ Currently, there are three main therapies for cardiovascular disease (CVD): statins (inhibitors of β-hydroxy β-methylglutaryl-CoA [HMG-CoA] reductase [HMGCR]),^4^^,^^5^ ezetimibe (inhibitors of Niemann-Pick C1-like 1 [NPC1L1]),^6^^,^^7^ and certain types of proprotein convertase subtilisin-kexin type 9 (PCSK9) inhibitors.^8^^,^^9^^,^^10^^,^^11^ While all three therapies, particularly statins, have demonstrated efficacy in clinical settings, a substantial number of patients still face residual risks despite intensive statin treatment or combination therapy.^12^^,^^13^^,^^14^ This highlights the urgent need for new and innovative therapeutic targets in the prevention and treatment of ASCVD.

ASGR1, the major subunit of ASGPR, is a highly conserved hepatocyte type II transmembrane glycoprotein across mammals.^15^^,^^16^ Since it was found by Morell and Ashwell in 1966, ASGPR has been defined as a key protein involved in recognizing and mediating the endocytosis and degradation of many circulating desialylated glycoproteins.^17^^,^^18^ A population genetic study identified that two loss-of-function mutations in ASGR1 (a noncoding 12-base-pair (bp) deletion (del 12) and p. W158X) were associated with lower non-HDL-C levels and reduced risks of cardiovascular diseases.^19^ Mice and pigs lacking ASGR1 can recapitulate the cholesterol-lowering effects features of ASCVD in humans with ASGR1 variants,^20^^,^^21^^,^^22^ which suggests that ASGR1 may functionally contribute to cholesterol metabolism regulation and be a potential target for ASCVD therapy. However, in the previous study, inhibition of ASGR1 in pigs caused mild to moderate liver injury, which raises concerns about the safety of ASGR1 inhibition as a target.^21^ It is necessary to further confirm the phenotype and explore whether ASGR1 inhibition causes liver damage in ASGR1-lacking pigs.

Atherosclerosis is a chronic inflammatory disease, increasing evidence suggests that the oxidation of LDL plays an integral role in both the initiation and the progression of this progress and triggers many putatively atherogenic events.^23^^,^^24^^,^^25^^,^^26^ Paraoxonases are a class of enzymes that can terminate lipid peroxidation at various cellular levels. There are three members in this family, PON1, PON2, and PON3, all three PON proteins can protect against LDL oxidation.^26^^,^^27^ Different from PON1 only expressed in the human liver, and PON3 in the liver and kidney, PON2 is a ubiquitously expressed type-2 transmembrane protein and it doesn’t associate with HDL in the circulation like PON1 and PON3.^28^ Mice lacking Pon2 fed with a high-fat diet developed significantly larger atherosclerotic lesions compared with wild-type mice, while elevated levels of PON2 significantly suppressed the progression of atheroma formation in six-month-old ApoE^−/−^ mice,^29^^,^^30^ indicating a protective role for PON2 in atherosclerosis.

In this study, we generated a new ASGR1 knockout allele in Bama minipigs using CRISPR/Cas9. Our pig models exhibited a reduced lipid profile without noticeable inflammation. RNA-seq analysis further confirmed the pivotal role of ASGR1 in cholesterol metabolism. Additionally, utilizing IP-MS, we characterized potential ASGR1-interacting proteins in HepG2 cells, revealing a series of novel interactions. We demonstrated the interaction between PON2 and ASGR1 for the first time, showcasing its ability to degrade ASGR1 both *in vitro* and *in vivo*. This interaction leads to a reduction in lipid levels in mice, accompanied by minimal inflammation. These results strongly advocate for the potential of ASGR1 inhibition as a promising strategy in CVD therapy, with PON2 emerging as a valuable and effective modulator.

## Results

### ASGR1^+/−^ pigs present a low lipid profile with no obvious inflammation

**Figure 1:**
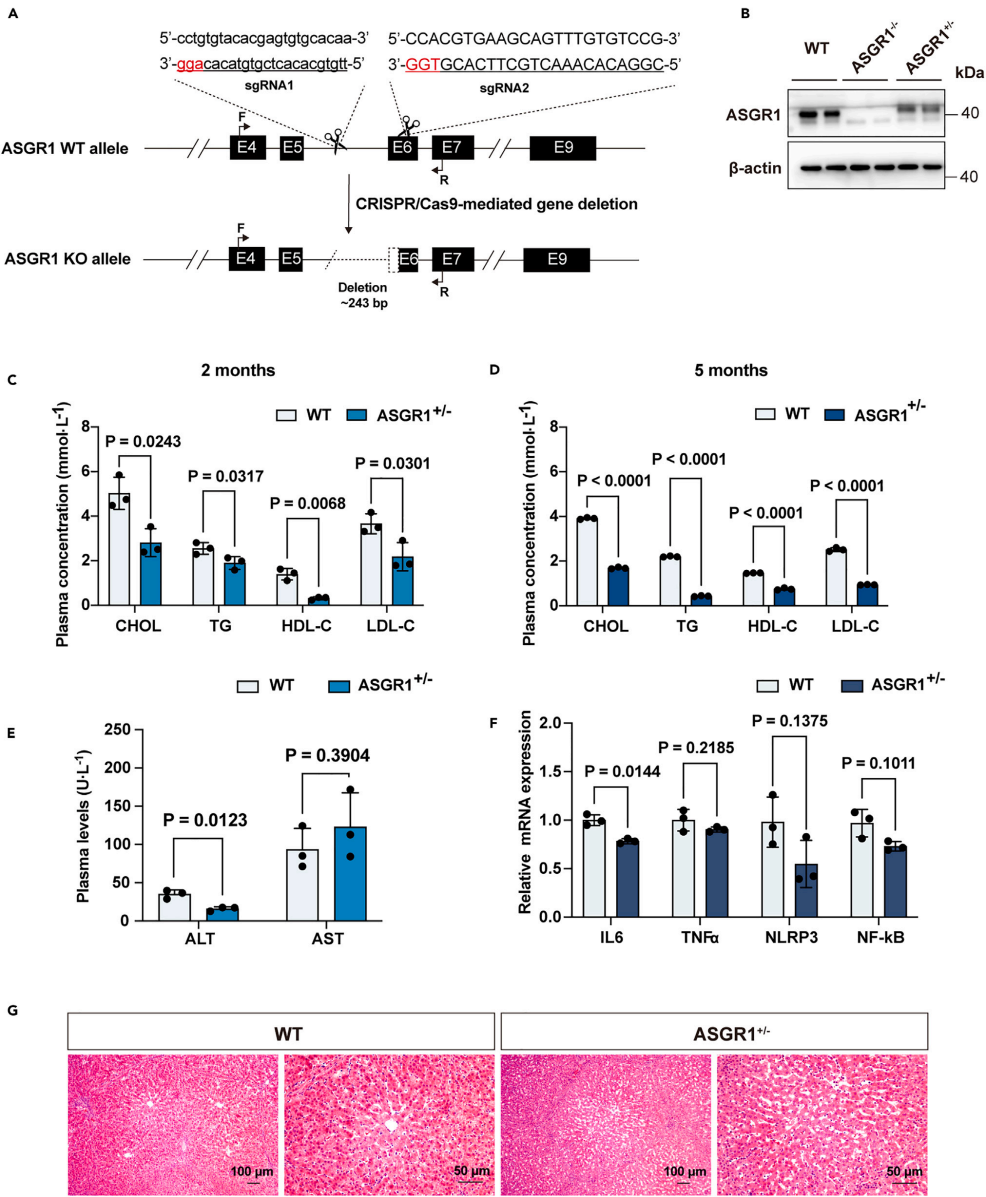
ASGR1^+/−^ pigs present a low lipid profile with no obvious inflammation (A) Schematic diagram of Cas9-sgRNA targeting sites of the pig ASGR1 locus. Two sgRNAs were used to target pig ASGR1 intron 5 (sgRNA1) and exon 6 (sgRNA2), potentially leading to a 243 bp deletion, with 25 bp deletion from exon 6. The sgRNA targeting sequences are underlined, and the PAM sequences are shown in red. Intronic sequences are represented in lowercase letters, while exonic sequences are in capital letters. (B) Immunoblotting of ASGR1 and internal control β-actin in the livers of WT, ASGR1^−/−^, and ASGR1^+/−^ pigs (*n* = 2). (C and D) TC, LDL-c, HDL-c, non-HDL-c, and TG contents in the serum of 2-month (C) and 5-month pigs (D) fasted overnight were analyzed (*n* = 3). (E) Plasma levels of ALT, AST in 2-month pigs (*n* = 3). (F) Relative mRNA expression of inflammation markers in livers of ASGR1^+/−^ pigs and WT controls (*n* = 3). Unpaired two-tailed Student’s t test calculated *p* values. All data are shown as the mean ± SEM. (G) H&E staining of liver sections from 2-month-old pigs fed a normal diet (Scale bars, 50 μm, and 100 μm). See also Figure S1.

Firstly, we regenerated ASGR1-knockout Bama miniature pigs with CRISPR/Cas9 gene editing. To increase the targeting efficiency, we designed two sgRNAs (Figure 1A). Transfection of the Cas9-sgRNAs targeting vectors into porcine fetal fibroblasts (PFFs) produced a total of 42 positive colonies, of which 9 carried biallelic modifications (−243 bp/−243 bp) and the others had monoallelic modifications (−243 bp/+) (Figure S1A). One biallelic-modified colony, #3, and one monoallelic-modified colony, #27, were selected and used as donor cells for somatic cell nuclear transfer. Two pregnant recipient sows received the transfer of a total of 520 reconstructed embryos, and three live-born male piglets were naturally delivered. Genotyping by Sanger sequencing identified that piglet #1 was ASGR1^−/−^ (homozygous) pig, and others were ASGR1^+/−^ (heterozygous) (Figure S1B). As predicted, ASGR1 was highly expressed in liver tissues of wild type (WT) pigs but was substantially decreased or undetectable in ASGR1^+/−^or ASGR1^−/−^ pigs, respectively (Figure 1B). We subsequently obtained 5 F1 live-born piglets, 4 of them were heterozygous for the −243 bp/+ mutation and F1-03 was female (Figures S1B and S1C). The appearance and behavior of ASGR1^+/−^ pigs did not show obvious abnormalities (Figure S1D), and no potential off-targets were shown in the generations of ASGR1^+/−^ pigs (Figure S1E).

Next, a biochemical analysis of the lipid profile was conducted to confirm the role of ASGR1 in cholesterol regulation. Results showed that the whole spectrum of lipids in serum was decreased in 2-month ASGR1^+/−^ pigs (Figure 1C), and it became significant at the age of 5 months (Figure 1D).

Since ASGR1 inhibition caused a reduced lipid profile in pigs, we examined whether its deficiency would cause an inflammatory response in the pigs. We analyzed the levels of clinical indicators (ALT and AST) in the serum of ASGR1^+/−^ pigs and found that there was no difference in AST expression level as compared to the WT pigs, while ALT levels were lower in ASGR1^+/−^ pigs (Figure 1E). We also examined the mRNA expression of inflammatory cytokines (IL6, TNFα, NLRP3, and NF-kB) and found that they were present at the same levels as in WT pigs (TNFα) or showed a downward trend (IL6, NLRP3, and NF-kB) (Figure 1F). Additionally, we observed no hepatic inflammation in ASGR1^+/−^ pigs (Figure 1G). These findings suggest that it may be a safe way to inhibit ASGR1 in pigs.

### Attenuated metabolic gene expression patterns in ASGR1^+/−^ pig livers

**Figure 2:**
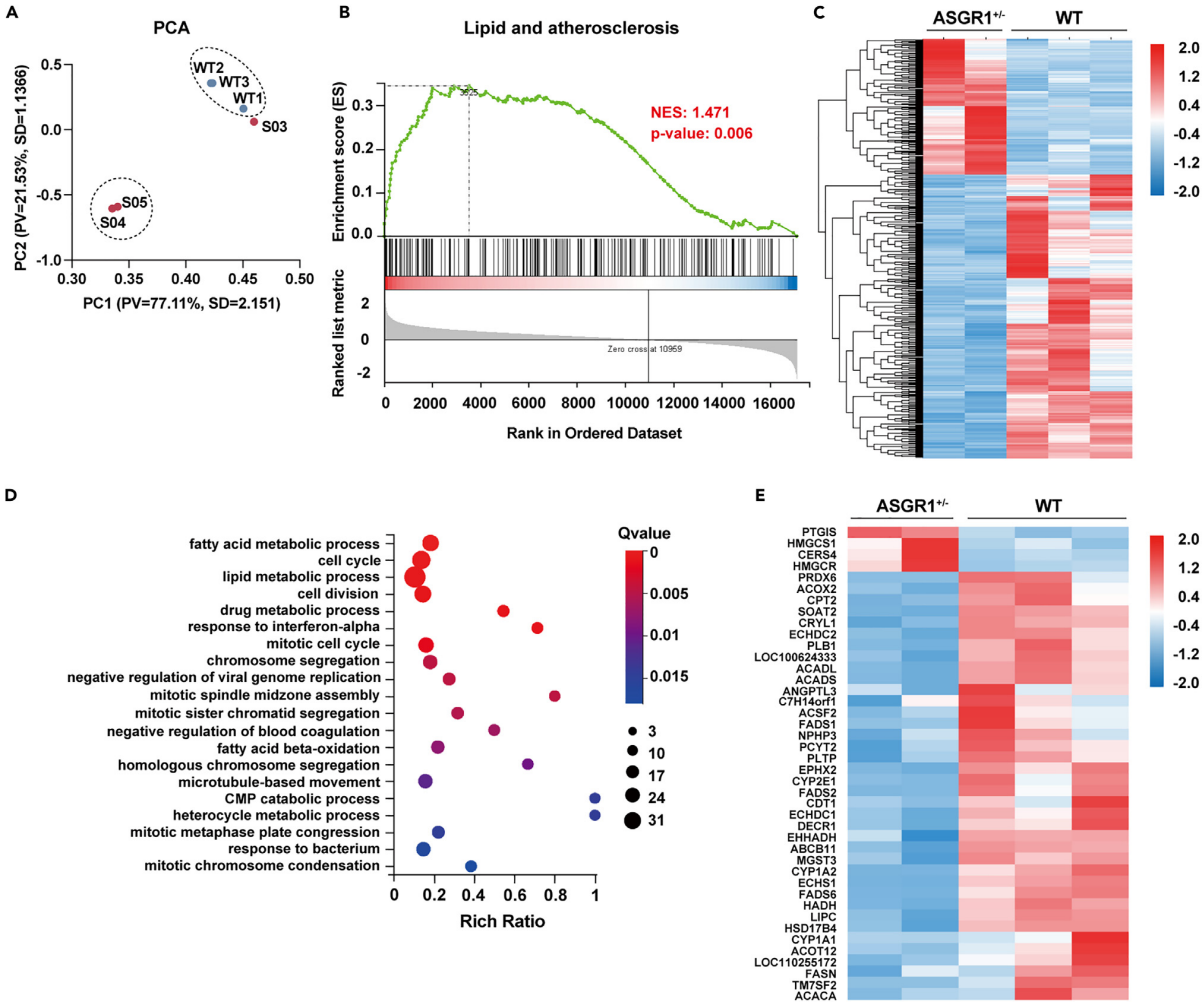
Attenuated metabolic gene expression patterns in livers of *ASGR1*^+/−^ pigs (A) PCA plots representing WT and ASGR1^+/−^ pigs based on transcriptome data (*n* = 3). (B) GSEA of gene sets for lipid and atherosclerosis. NES, normalized enrichment score. (C) Heatmap shows hierarchical clustering of differentially expressed genes (DEGs) in ASGR1^+/−^ pigs. Values are column-scaled to show expression level. (D) The DEGs were subjected to GO analysis, and the dot plot shows the most significantly enriched pathways. The color of the dots represents the q value, and the size of the dots represents the number of differentially expressed transcripts. (E) Heatmap shows hierarchical clustering of differentially expressed genes in the metabolic pathway. Values are column-scaled to show expression level.

To gain a better understanding of ASGR1 gene expression patterns, we conducted RNA-seq analysis on liver tissues of WT and ASGR1^+/−^ pigs. Principal-component analysis (PCA) revealed a clear classification of the two groups of liver samples, except for S03, which was subsequently excluded from subsequent analyses (Figure 2A). In summary, these results indicate distinct differences in their gene expression landscapes. Gene set enrichment analyses (GSEAs) showed the lipid and atherosclerosis pathway was significantly enriched in ASGR1^+/−^pig livers (NES = 1.471, *p* = 0.006) (Figure 2B). Further examination of the differentially expressed genes (DEGs) identified 536 genes with significant changes (|log2FC| ≥ 1, q value ≤0.05), among which 362 genes were downregulated, while 174 genes were upregulated (Figure 2C). These DEGs were subjected to gene ontology (GO) biological process analysis, which identified significant enrichment in metabolic pathways such as fatty acid metabolic processes, lipid metabolic processes, and fatty acid beta-oxidation (Figure 2D). These findings indicated the pivotal role of ASGR1 in metabolic functions. Then we selected the 42 metabolism-related genes for heatmap construction. The results showed that most genes were downregulated in ASGR1^+/−^ pigs. The data indicate that ASGR1 may play a significant physiological part in cholesterol homeostasis.

### Identification of ASGR1-interacting proteins in HepG2 cells

**Figure 3:**
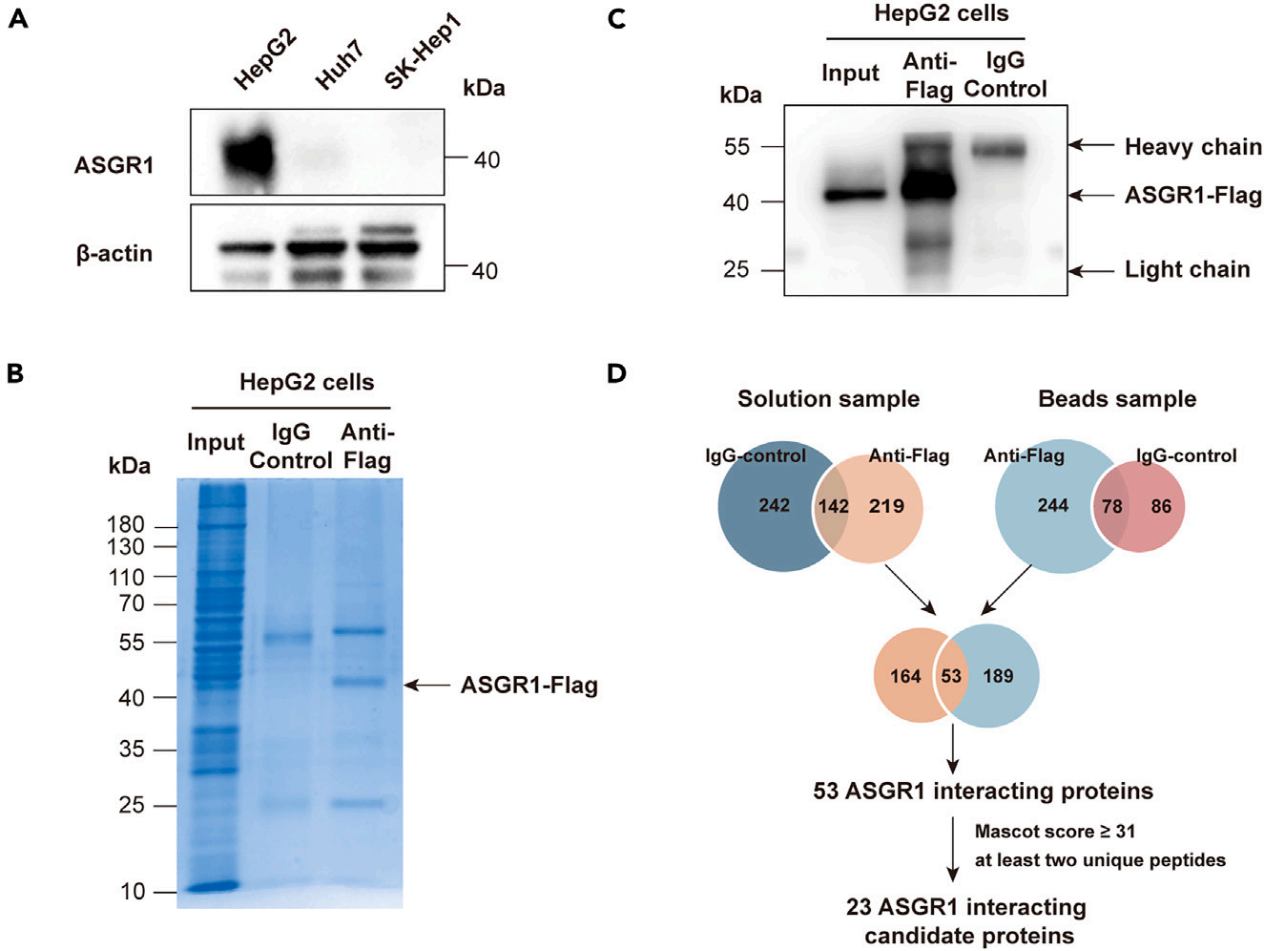
IP-MS analysis of ASGR1-interacting complex (A) Immunoblotting analysis of the expression of ASGR1 in three human liver cells. (B) SDS-PAGE band pattern of immunoprecipitated proteins in HepG2 cells with anti-Flag antibody versus isotype-controlled IgG. (C) Immunoblotting analysis to confirm the presence of ASGR1 in the immunoprecipitated samples. (D) Venn diagram illustrating the number of both specific and nonspecific ASGR1 interactors. Only peptides with a Mascot score ≥31 were counted as identified, and each confident protein identification involves at least two unique peptides. See also Table S2.

To identify the new proteins that bind and degrade the ASGR1 level, we performed an immunoprecipitation technique combined with mass spectrometry (IP-MS) to identify ASGR1-interacting proteins in HepG2 cells. We first examined ASGR1 expression in several liver cell lines by western blot and observed significantly higher expression of ASGR1 in HepG2 compared to other liver cell lines (Figure 3A). Thus, we used IP-MS to isolate ASGR1 and identify its interacting partners in HepG2 cells. To enhance the enrichment levels, we constructed ASGR1 overexpressed HepG2 cells containing a C-Flag tag. We found a distinct band at 40 kDa only in anti-Flag-IP samples of HepG2 cells (Figure 3B). Western blot analysis validated that the ASGR1 band was present only in the anti-Flag-IP sample, which indicates a successful ASGR1 pull-down by IP (Figure 3C). Each lane of the immunoprecipitated proteins derived from anti-Flag or IgG control was excised and underwent tryptic digestion and identification by LC-MS/MS. In total, 603 and 408 proteins were identified in the anti-Flag-IP solution and beads sample in HepG2 cells, respectively.

Proteins detected in the IgG-control samples were deemed nonspecific and subsequently excluded from further analysis. By subtracting 384 and 164 nonspecific binding proteins, we identified 219 and 244 potential ASGR1-interacting partners in HepG2 cells, respectively. Among the identified proteins, 53 appeared in both the solution and beads samples (Figure 3D). “Mascot score” provides an acceptance threshold with false identification probability at a confidence level of 0.05. To reduce the probability of false peptide identification, only peptides with Mascot scores ≥31 were counted as identified, and each confident protein identification involves at least two unique peptides. Finally, we obtained 23 candidate proteins, all 23 ASGR1-interacting proteins are listed in Table S2 and classified according to the description of proteins, percentage of coverage, and peptide-spectrum matches (PSM, the total number of identified peptide spectra matched for the protein).

### Validation of the association between ASGR1 and its novel interacting partners

**Figure 4:**
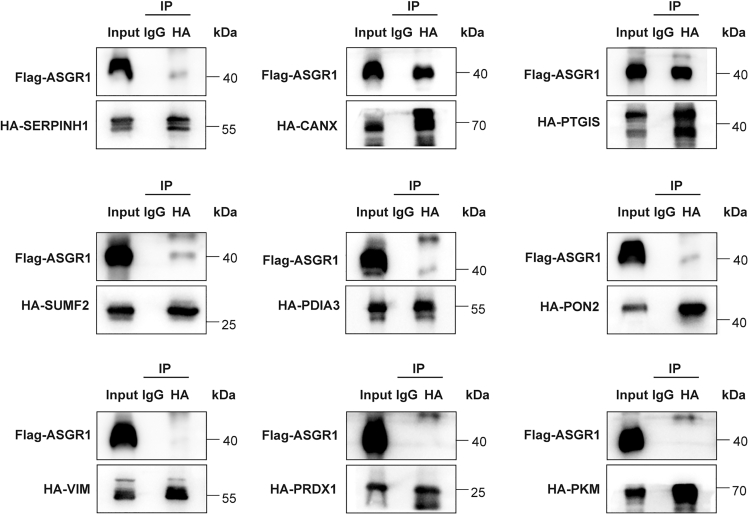
Validation of the selected ASGR1-interacting proteins Coimmunoprecipitation of the candidate proteins and ASGR1 in HEK293T cells. Pull-downs with HA antibodies were analyzed by western blot for Flag (ASGR1) and HA (candidate proteins). Isotype-controlled rabbit “IgG” as a negative control. “Input” represents 10% of the original material subjected to coimmunoprecipitation. See also Figure S2.

Among the 23 proteins identified in HepG2 cells, the majority were not previously recognized as interacting partners of ASGR1. We selected 9 proteins for validation. The proteins that were chosen have been selected based on their importance in metabolic processes. Subsequently, we endeavored to validate the interaction between ASGR1 and these selected proteins. All candidate genes were cloned into vectors featuring a C-HA tag, while the ASGR1 gene was cloned into a vector containing a C-FLAG tag (Figure S2A). Subsequently, equal amounts of pEF1a-ASGR1-3 × Flag-IRES-GFP and pEF1a-Candidates-3 × HA-SV40-Puro plasmids were co-expressed transiently in HEK293T cells, and the control group was transfected pEF1a-ASGR1-3 × Flag-IRES-GFP and pEF1a-3 × HA-SV40-Puro empty vector (Figure S2B). CoIP was conducted in whole-cell extracts using HA-tag antibodies and matched normal IgG as a negative control. Among the 9 selected proteins, we confirmed the binding of 6 proteins to ASGR1. However, three proteins (VIM, PKM, PRDX1) showed no obvious interaction with ASGR1 (Figure 4). These findings instill confidence in the validity of many other ASGR1 interactors identified through the MS screen.

Next, we explored how the new proteins in the circulation system binding to ASGR1 regulate its levels. When ASGR1 and candidate proteins were co-overexpressed in equal amounts within HEK293T cells, PON2 was observed to exert the most pronounced inhibitory effect on cellular green fluorescence compared to the control group. This observation suggested that PON2 could potentially exert an inhibitory effect on ASGR1 (Figure S2B). To confirm this hypothesis, we performed a transfection experiment using HepG2 cells, which naturally express ASGR1. Specifically, we introduced equal quantities of the pEF1a-Candidates-3 × HA-SV40-puro plasmids into these cells. 48 h after transfection, we collected all the cells and assessed the expression of ASGR1 at both mRNA and protein levels. The results showed that, compared to the other candidate interacting proteins, PON2 can significantly suppress the expression of ASGR1 at the protein level, rather than at the transcriptional level in the HepG2 cells (Figures S2C and S2D). Subsequently, we conducted further investigation into the regulatory effects of PON2 on ASGR1.

### PON2 interacts with ASGR1 and degrades its protein level in a dose-dependent manner

**Figure 5:**
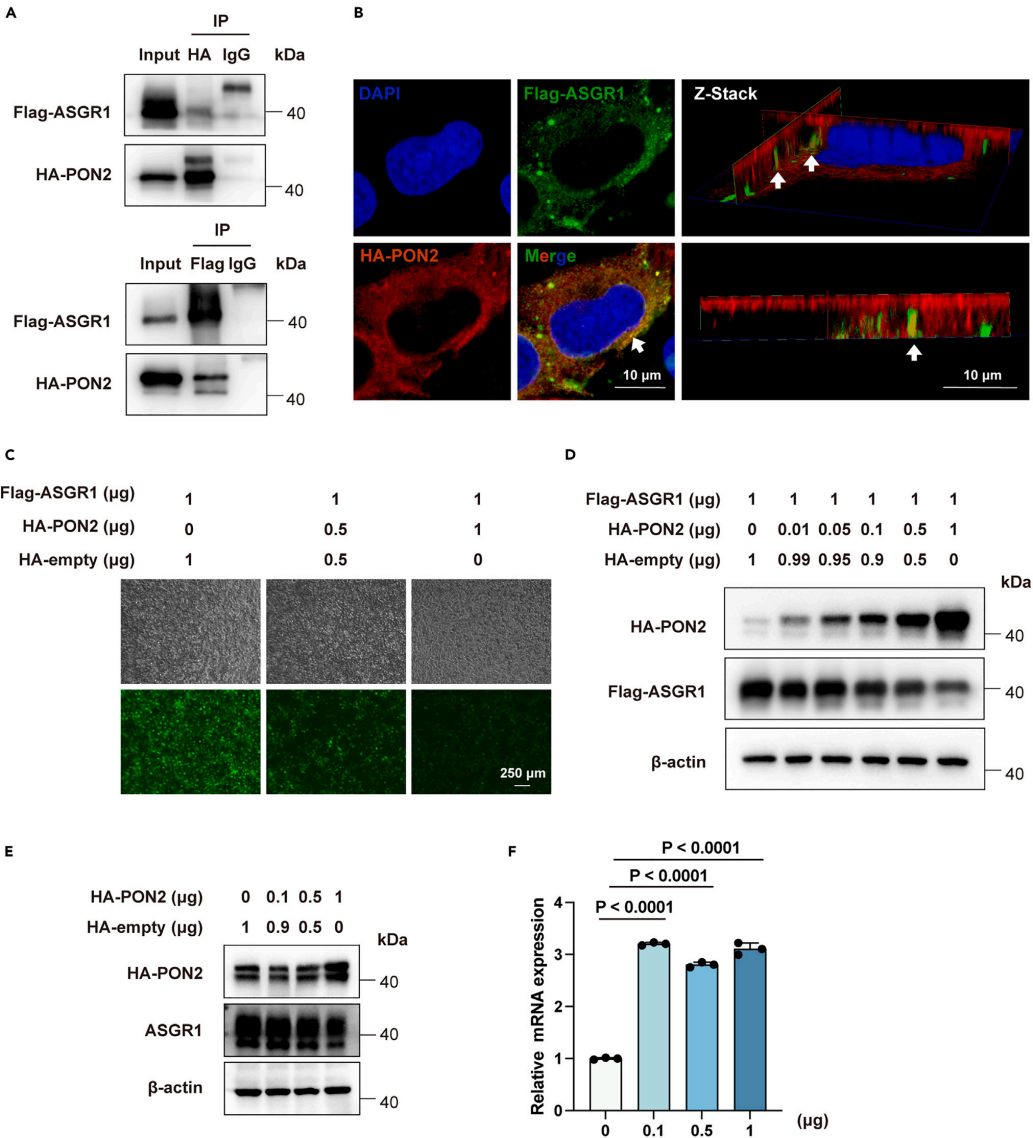
PON2 interacts with ASGR1 and degrades its protein level in a dose-dependent manner (A) Coimmunoprecipitation from HEK293T cells transfected with HA-tagged PON2 and Flag-tagged ASGR1. Pull-downs with HA antibody (up panel) or Flag antibody (down panel) were analyzed by western blot for PON2 and ASGR1. “Input” represents 10% of the original material subjected to coimmunoprecipitation. Data are representative of two independent experiments. (B) Immunofluorescence microscopy of PON2 (red signal) and ASGR1 (green signal) in Huh7. White arrows, co-localizing dots (Scale bars, 10 μm). (C) HEK293T cells were transfected with ASGR1 and different doses of PON2, and additional pEF1a-3 × HA-SV40-Puro empty vector was transfected to ensure consistency in the total amount of plasmid DNA introduced into the cells (Scale bars, 250 μm). (D) Immunoblotting analysis of the protein levels of PON2 and ASGR1 in the HEK293T cells. (E) Immunoblotting analysis of the protein levels of PON2 and ASGR1 in the HepG2 cells. (F) Expression of ASGR1 in HepG2 cells measured by RT-qPCR (*n* = 3). All values are presented as mean ± SEM. *p* values were calculated by unpaired two-tailed Student’s t test. See also Figure S3.

We validated the ASGR1-PON2 interaction by co-IP, and the results demonstrated that interaction between the two proteins could be detected regardless of whether the Flag or HA tag was used as bait (Figure 5A). Immunofluorescence assays in Huh7 cells further confirmed their colocalization (Figure 5B). We found that the degradation effect of PON2 on ASGR1 protein levels is dose-dependent in HEK293T cells (Figures 5C and 5D) and HepG2 cells (Figure 5E), while it couldn’t decrease ASGR1 at the transcriptional level in the HepG2 cells (Figure 5F). We extended our examination to murine Pon2 to assess its potential down-regulation of murine Asgr1. The results demonstrated that Pon2 downregulated Asgr1 protein level in a dose-dependent manner (Figures S3A and S3B), highlighting a conserved function of Pon2 in downregulating Asgr1.

After establishing the inhibitory effect of PON2 on the ASGR1 protein level, we intend to explore the specific sequence within PON2 that is responsible for its function. Given that we did not find any reports about the structural domains of PON2, we roughly divided it into three segments for deletional experiments (Figure S4A) and co-expressed these truncations-HA plasmids and ASGR1-FLAG in HEK293T cells (Figure S4B). The full-length PON2, truncations PΔ2 (Δ123–231) and PΔ3 (Δ232–354) were coprecipitated by ASGR1 and demonstrated a strong potency in lowering ASGR1 protein expression, indicating that these variants contain the motifs responsible for ASGR1 interaction and degradation (Figure S4C). In contrast, the PON2 truncations P1(Δ1–122) failed to bind ASGR1 or induce its degradation. Deletion of 2–122 in PON2 abolished its activity of inducing ASGR1 degradation. We also expressed the region (a.a. 1–122) of PON2 (HA-PON2-Z1) and Flag-ASGR1 at the same time in the HEK293T and found that this region can interact with ASGR1 and decrease its protein level in a dose-dependent manner (Figures S4D and S4E). Collectively, these results suggest that the region (a.a. 1–122) of PON2 is required for interacting with and degrading ASGR1.

### Overexpressed PON2 reduces ASGR1 and lipid levels *in vivo*

**Figure 6:**
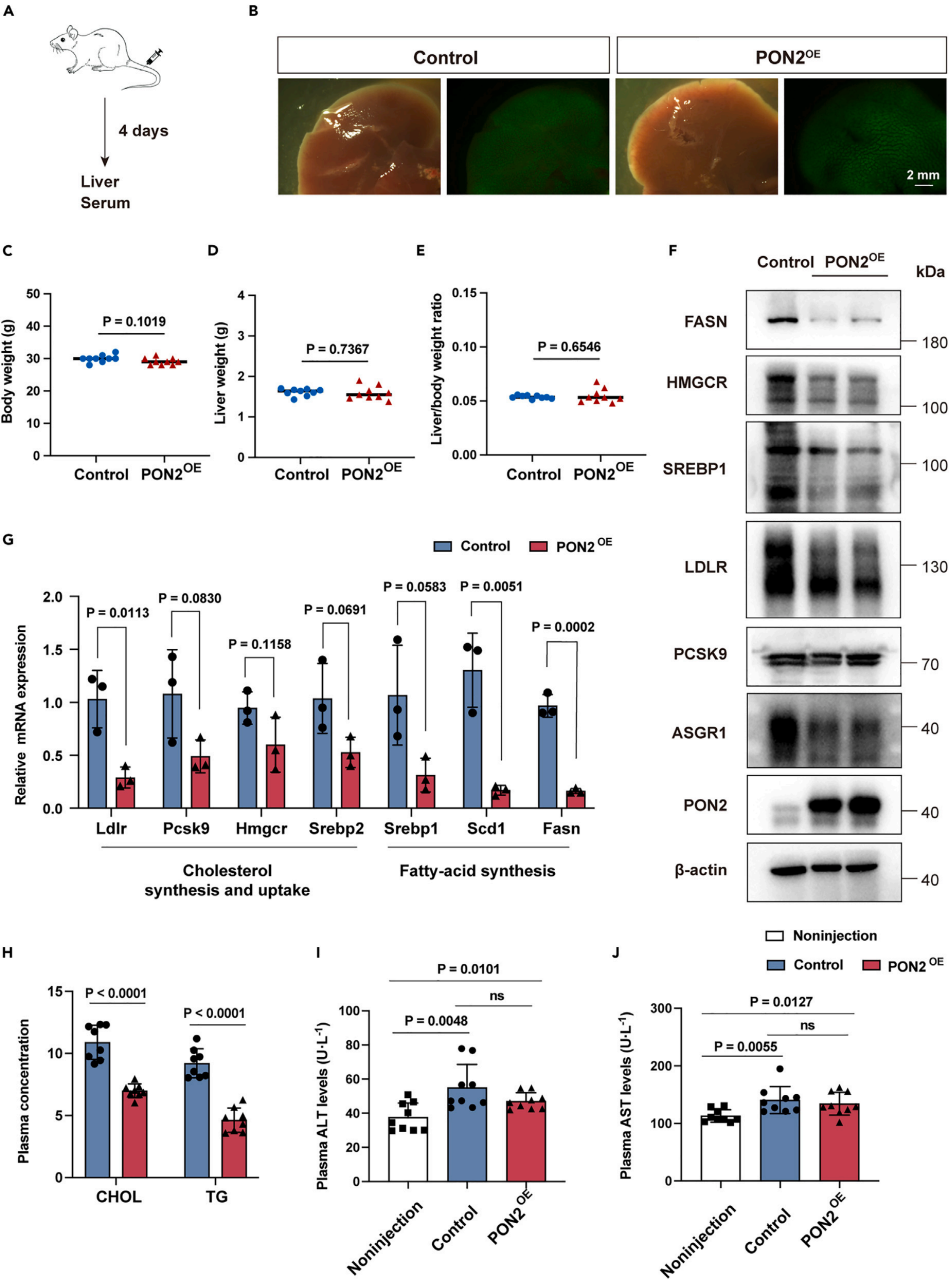
PON2 reduces ASGR1 and lipid levels *in vivo* (A) Injection scheme. (B) Mice liver tissues under the fluorescent microscope (Scale bars, 2 mm). (C) Body weight. (D) Liver weight. (E) Liver/body weight ratio (*n* = 9). (F) Immunoblotting analysis of PON2^OE^ mice livers. (G) Expression of the metabolism-related genes in mouse liver measured by RT-qPCR (*n* = 3). (H) Plasma levels of CHOL and TG (*n* = 8). (I) Plasma levels of ALT (*n* = 9). (J) Plasma levels of AST (*n* = 9). All values are presented as mean ± SEM. *p* values were calculated by unpaired two-tailed Student’s t test.

Since several studies have demonstrated that PON2 has a positive effect on atherosclerosis, we aimed to investigate the effect of PON2 on endogenous ASGR1 expression and further explore the follow-up effects of this impact. To determine whether PON2 has a comparable effect *in vivo*, plasmids pEF1a-ASGR1-3 × Flag-IRES-GFP, and pCAG-PBase were delivered to the mouse liver through hydrodynamic tail-vein injection, what’s more, the control group injected with pEF1a-3 × HA-SV40-Puro plasmid, while the PON2 group injected with pEF1a-PON2-3 × HA-SV40-Puro plasmid at the same time (Figure 6A). After 4 days of plasmid injection, we directly dissociated the GFP-labeled liver tissues under the fluorescent microscope (Figure 6B). No differences were observed among the three groups in terms of body weight (Figure 6C), liver weight (Figure 6D), and the liver-to-body weight ratio (Figure 6E), indicating no changes between the control and PON2 groups. Western blot analysis demonstrated successful expression of PON2 compared to the control, accompanied by decreased expression of ASGR1, FASN, and HMGCR (Figure 6F). The mRNA levels of genes related to cholesterol synthesis and uptake and fatty acid synthesis all exhibited a decreased trend (Figure 6G). Notably, the mice in the PON2 group showed significantly reduced levels of serum cholesterol (CHOL) and triglyceride (TG) levels (Figure 6H). In contrast, the serum levels of ALT and AST did not differ from the control group. However, the levels of ALT and AST were elevated in the group subjected to hydrodynamic tail-vein injection when compared to the noninjected WT group (Figures 6I and 6J). These results suggest that PON2 serves as a promising regulator of ASGR1 and holds potential therapeutic implications.

## Discussion

Since the first report indicated that heterozygous carriers of the early termination LOF del12 mutation in the ASGR1 gene (1 in 120 persons in the Icelandic study) had lower plasma levels of non-HDL cholesterol and CVD risk than noncarriers,^20^ the function and mechanism of ASGR1 in cholesterol homeostasis has been explored with great enthusiasm. Up to now, ASGR1-lacking mice and pigs have been constructed and recapitulated the cholesterol-lowering effects in humans successfully, which means ASGR1 could be a potential target for treating CVD.^20^^,^^21^^,^^22^ However, the Asgr1-knockout mice showed normal growth and development, with no significant difference in body weight and hepatic histology among different genotypes mice, while pigs lacking ASGR1 experienced different degrees of hepatic injury, causing concerns about its safety as a therapeutic target. In our study, we regenerated a new knockout allele of ASGR1 in pigs using CRISPR/Cas9. In the two-month-old ASGR1^+/−^ pigs, there was a noticeable trend toward decreased levels of serum CHOL, TG, and LDL-C, which became statistically significant by the age of five months. This contrasts with a previous study, which reported markedly lower lipid levels in pigs only after at least ten months of age. In addition, we examined the expression of the inflammation markers, the serum AST and ALT levels, and the histological evaluation of liver sections, all the results showed our pigs had no obvious liver damage, while the ASGR1-knockout pigs in the previous study showed mild to moderate liver injury, with the elevated inflammatory cytokines and fibrotic markers. The discrepancies observed between our study and previous ASGR1-knockout pig models may be attributed to several factors, including the distinct genetic backgrounds of the pigs, variations in sgRNA design, and potential off-target mutations. Each pig model, regardless of the genetic modification, carries a unique genetic background that can influence the phenotype expressed after the knockout. Genetic variations outside the ASGR1 gene, such as polymorphisms in modifier genes, can alter the physiological response to the knockout. What’s more, the design of the sgRNAs is critical to the specificity and efficiency of the CRISPR/Cas9 system, and the off-target mutations induced by Cas9 also cannot be ignored. The sgRNAs utilized in our study were designed for generating ASGR1-knockout pigs previously, and we examined those pigs by NT-seq and found no off-target events induced by Cas9,^31^ which means our sgRNAs design is successful and the pigs in our study are reliable for exploring the function and regulation network of ASGR1. All our results demonstrated that ASGR1 inhibition could be a safe way in pigs.

Mechanically, there are primarily two approaches to regulate cholesterol homeostasis: inhibiting cholesterol synthesis and enhancing cholesterol clearance. We examined the expression of HMGCR and LDLR, two key proteins in these processes, and the protein level of HMGCR was downregulated in the liver of ASGR1^+/−^ pigs. Surprisingly, however, LDLR was also downregulated. We also examined the expression of PCSK9, and there is no significant difference between the two groups (data not shown). These experiments demonstrate that ASGR1 may not regulate non-LDL-C through the PCSK9-LDLR pathway and indicate other operative mechanisms requiring exploration. Studies in Asgr1-knockout mice showed that downregulating of ASGR1 led to a decrease in nuclear SREBPs (nSREBPs), with one showing it anchored SREBP1 to the endoplasmic reticulum via increasing the expression of the insulin-induced gene 1 (INSIG1),^20^ while the other suggested its downregulation of mammalian target of rapamycin complex 1 (mTORC1) and the upregulation of AMP-activated protein kinase (AMPK), which eventually inhibited SREBP1 expression and activation.^22^ Since SREBPs function as transcription factors via negative feedback control to regulate lipogenesis and uptake and biosynthesis of cholesterol, and our transcriptome analysis showed that ASGR1 knockout led to decreased expression of a group of genes related to lipid metabolism, we detected the downregulated expression of SREBP1 and SREBP2, indicating that ASGR1 does play a role in cholesterol metabolism (data not shown).

Compelling evidence suggests that the AMPK-mTOR-autophagy pathway is important in atherosclerosis.^32^^,^^33^^,^^34^ Recent findings indicate that AMPK controls autophagy through the downstream mTORC1-ULK1 signaling pathway, which is accountable for initiating autophagy,^35^ and it also induces autophagy through the direct phosphorylation of ULK1 and beclin-1.^36^ In the Asgr1 knockout mice, the expression of *p*-AMPK, *p*-ULK1, and p-Raptor was significantly elevated,^22^ which could potentially activate downstream autophagy pathways. Therefore, we examined the expression of key molecules in the autophagy pathway in ASGR1 knockout pig liver tissues. As expected, the downregulation of ASGR1 in pigs activated the autophagy pathway (data not shown). Further experiments are needed to demonstrate the beneficial impact of ASGR1 inhibition-induced autophagy on its protective effect against atherosclerosis.

ASGR1 holds promise as a therapeutic target for the clinical management of hypercholesterolemia. Being a receptor localized on hepatocyte surfaces, ASGR1 offers a tractable avenue for the development of therapeutic interventions. Specifically, it is feasible to identify ligand analogs or small-molecule compounds capable of competitively binding to and inactivating ASGR1, thereby providing a potential strategy for cholesterol-lowering treatments. At present, there are still relatively few reports on inhibitors of ASGR1, and only a few of its endogenous interacting proteins have been identified. In this study, we developed an immunoprecipitation combined with mass spectrometry (IP-MS) method to identify endogenous interacting proteins of ASGR1 in HepG2 cells. We obtained a total of 23 credible candidate interacting proteins, and among the functionally relevant 9 candidate proteins, we preliminarily identified 6 proteins that interact with ASGR1, none of which have been reported before. We failed to detect any known ASGR1 interactors except for GPX8 within the beads sample.^37^ Several factors could contribute to the absence of other potential interactors. Firstly, the protein-protein interactions of interest are not expressed or are not detectable in the HepG2 cell line used in our experiments. Secondly, some interactions with ASGR1 might be transient, making them difficult to capture under the conditions of our assay. Thirdly, the protein might have a lower affinity toward ASGR1. What’s more, the over-filtering of the data might have inadvertently excluded some specific interaction partners. Ideally, proteins that interact nonspecifically with the IgG control should be disregarded as they are not considered true interactors. However, there is a possibility that some genuine interaction partners may also exhibit nonspecific binding to IgG or beads, leading to their removal from the analysis. Further research revealed that PON2 can dose-dependently decrease the ASGR1 protein level, and this effect is evolutionarily conserved across species. Our study is the first to describe the interaction between ASGR1 and PON2, and the subsequent metabolic effects. This interaction appears to be a novel post-translational regulatory mechanism that could be targeted for therapeutic intervention. In the future, the detailed mechanistic understanding of PON2’s effects on ASGR1, coupled with the *in vivo* evidence of its therapeutic potential, may open up new possibilities for the development of targeted therapies for metabolic diseases, particularly hypercholesterolemia.

In conclusion, our results showed that inhibition of ASGR1 in pigs may be a safe and effective way for lipid reduction, and the role of ASGR1 in the APMK-autophagy pathway is worth exploring, which may uncover the additional protective effect against atherosclerosis of ASGR1. In addition, the interacting proteins identified in our study provide a better way to understand the function of ASGR1, and PON2 is a hopeful target to inhibit ASGR1 and prevent atherosclerosis.

### Limitations of the study

In this study, we generated ASGR1 knockout pigs, and compared to WT pigs, the ASGR1^+/−^ pigs exhibited a lower lipid profile and no obvious inflammation. Although we identified the phenotype of ASGR1 haploinsufficiency *in vivo*, the actual mechanism of ASGR1 regulating lipogenesis and inflammation was not investigated. What’s more, due to the difficulty of the experiments on large animals, the concentrations of serum CHOL, TG, HDL-C, LDL-C, ALT, and AST were detected in only three animals per group, which may account for the deviations observed in the results. We also found that PON2 interacts with ASGR1 and inhibits it *in vitro* and *in vivo*, which reduces the concentrations of serum CHOL and TG, but the actual mechanism of PON2 regulating ASGR1 remains unclear. Additionally, the *in vivo* effects of the PON2 variant (1-122aa) on modulating ASGR1 expression require further investigation.

## STAR★Methods

### Key resources table

**Table:** 

REAGENT or RESOURCE	SOURCE	IDENTIFIER
**Antibodies**		

Monoclonal anti-Flag	Sigma-Aldrich	Cat# F1804; RRID: AB_262044
Anti-HA	Cell signaling technology	Cat# C29F4
Mouse monoclonal anti-Actin	Beyotime	Cat# AA128; RRID: AB_2861213
ASGR1 Antibody	Proteintech	Cat# CL594-11739; RRID: AB_2919773
Anti-PON2	Abcam	Cat# ab183710
Anti-PCSK9	Beyotime	Cat# AF7692
FASN Rabbit mAb	ABclonal	Cat# A19050; RRID: AB_2862543
Anti-LDLR	ABclonal	Cat# A20808
HMGCR Polyclonal Antibody	ABclonal	Cat# A16875; RRID: AB_2769811
Anti-SREBP1	ABclonal	Cat# A25305
Mouse IgG	Beyotime	Cat# A7028; RRID: AB_2909433
HRP-labeled Goat anti-Rabbit IgG (H + L)	Beyotime	Cat# A0208; RRID: AB_2892644
Horseradish-labeled goat anti-mouse IgG (H + L)	Beyotime	Cat# A0216; RRID: AB_2860575
Goat anti-Mouse IgG (H+L) Cross-Adsorbed Secondary Antibody, Alexa Fluor 488	Thermo Fisher Scientific	Cat# A32723TR; RRID: AB_2866489
Goat anti-Rabbit IgG (H+L) Cross-Adsorbed Secondary Antibody, Alexa Fluor 594	Thermo Fisher Scientific	Cat # A-11037; RRID: AB_2534095

**Biological samples**		

ASGR1^+/-^ and WT pigs ear tissue	This paper	N/A
ASGR1^+/-^ and WT pigs liver tissue	This paper	N/A
ASGR1^+/-^ and WT pigs plasma	This paper	N/A
PON2^OE^ and control mice liver tissue	This paper	N/A
PON2^OE^, control and WT mice plasma	This paper	N/A

**Chemicals, peptides, and recombinant proteins**		

Fetal Bovine Serum (FBS)	Gibco	Cat # 10099
Dulbecco’s modified Eagle’s medium (DMEM)	Gibco	Cat # 12100061
Dulbecco’s phosphate-buffered saline (DPBS)	Gibco	Cat # D2650
0.25% Typsin-EDTA	Gibco	Cat # 25200072
penicillin/streptomycin	Thermo Fisher Scientific	Cat # 15140122
nonessential amino acid	Gibco	Cat # 11140050
PMSF	Beyotime	Cat # ST506
LentiFit	HanBio	Cat # HB-LIF-1000
Pierce Protein A/G Magnetic Beads	Thermo Fisher Scientific	Cat # 88803

**Critical commercial assays**		

ABScript III RT Master Mix for qPCR	ABclonal	Cat # RK20429
2 × RealStar Fast SYBR qPCR Mix	GenStar	Cat # A301

**Deposited data**		

Raw data of RNA sequencing	This paper	GEO: GSE268910
Raw data of mass spectrometry	This paper	iProX: PXD052849
Raw data of Western blotting	This paper	Mendeley Data: https://doi.org/10.17632/n5nthvrf3p.1

**Experimental models: Cell lines**		

HEK293T	ATCC	Cat # CRL-3216 RRID: CVCL_0063
HepG2	ATCC	Cat # HB-8065 RRID: CVCL_0027
Huh7	Procell	Cat # CL0120 RRID: CVCL_0336
PFF	This paper	N/A

**Experimental models: Organisms/strains**		

Mouse: CD-1 (ICR)	Vital River	RRID: MGI: 5649797
Pig: ASGR1 knockout: Bama minipigs	This paper	N/A

**Oligonucleotides**		

See Table S1	This paper	N/A

**Recombinant DNA**		

Plasmid: pM3-Cas9	Lu et al.^38^	N/A
Plasmid: pCRISPR-sg6	Xu et al.^39^	N/A
Plasmid: pPB-hNRAS^G12V^	Xu et al.^39^	N/A
Plasmid: pMax2-Cas9-GFP	This paper	N/A
Plasmid: pASGR1-sgRNA1	This paper	N/A
Plasmid: pASGR1-sgRNA1	This paper	N/A
Plasmid: pEF1a-ASGR1-IRES-GFP	This paper	N/A
Plasmid: pEF1a- ASGR1-3 × Flag - IRES-GFP	This paper	N/A
Plasmid: pEF1a-3 × HA-SV40-Puro	This paper	N/A
Plasmid: pEF1a-Candidates-3 × HA -SV40-Puro	This paper	N/A
Plasmid: pCAG-PBase	This paper	N/A

**Software and algorithms**		

Image J	Open source	N/A
Prism 9	Graphpad Software	N/A

**Other**		

Roche LightCycler 480 Real Time PCR System	Roche	RRID: SCR_018626
Nucleofector 2b Device	Lonza	RRID: SCR_022262
Leica Stellaris 5 microscope	Leica	RRID: SCR_024663

### Resource availability

#### Lead contact

Further information and requests for resources and reagents should be directed to and will be fulfilled by the Lead Contact, Sen Wu (swu@cau.edu.cn).

#### Materials availability

Unique materials generated in this study are available upon completing materials transfer agreement.

#### Data and code availability

• RNA-seq data have been deposited at GEO and are publicly available as of the date of publication. Accession numbers are listed in the key resources table. The MS data have been deposited to the ProteomeXchange Consortium via the iProX repository and are publicly available as of the date of publication. Accession numbers are listed in the key resources table. Original western blot images have been deposited at Mendeley and are publicly available as of the date of publication. The DOI is listed in the key resources table.
• This paper does not report original code.
• Any additional information required to reanalyze the data reported in this paper is available from the lead contact upon request.

### Experimental model and study participant details

#### Animals model

All pig lines were of Bama minipigs’ genetic background or their offspring. Pigs aged about 8−20 weeks were used for experiments, and the age difference of the pigs was no more than 1 week for the same experiment. All pigs generated in our study were male except for F1-03. Pigs were housed in a controlled environment (12−hour daylight cycle), with free access to normal food and water.

CD-1 (ICR) mice were obtained from Vital River Laboratory Animal Technology (Beijing, China). All mice were 4 weeks old male and used for Hydrodynamic tail vein injection. All animals were fasted for 12h (overnight) before scarification, and blood was collected to isolate the serum for biochemical analysis. Animal experiments in this study were performed following the guide of the Animal Welfare Committee of China Agricultural University AW03111202-3-1.

#### Cell culture

HEK 293T cells, PFFs, HepG2, and Huh7 were cultured with DMEM (Gibco) supplemented with 10% FBS (Gibco), 1% nonessential amino acid (Gibco) and 1% penicillin/streptomycin (Thermo Fisher Scientific) in an incubator at 37°C with 5% CO_2_.

### Method details

#### Plasmid construction and transfection

The sgRNAs targeting porcine endogenous ASGR1 were designed and listed in Table S1. Oligonucleotides for sgRNA templates were synthesized, annealed, and inserted into the *Bbs*I site of pCRISPR-sg6.^38^ To generate the Cas9-expressing plasmid pMax2-Cas9-GFP, the pM3-Cas9 plasmid^39^ was digested with *Fse*I. The F2A-EGFP fragment was PCR amplified and inserted into the *Fes*I-digested pM3-Cas9 backbone through Gibson assembly. The human ASGR1-CDS sequence was synthesized by General Biol and then inserted into the *Nhe*I and *Bam*HI-digested pPB-hNRAS^G12V^ to generate the ASGR1 overexpression plasmid pEF1a-ASGR1-IRES-GFP. To generate the pEF1a-ASGR1-3 × Flag-IRES-GFP, the pEF1a-ASGR1-IRES-GFP was digested with *Sbf*I and fused a 3 × Flag fragment in the C terminal of ASGR1-CDS. The candidates’ CDS sequences were amplified from a template of HepG2 cells cDNA and then inserted into the *Nhe*I-digested pEF1a-3 × HA-SV40-Puro backbone through T4 ligase. Mouse Asgr1- and Pon2-CDS were amplified from the template of mouse liver cDNA. Truncations of PON2 were amplified from the plasmid pEF1a-PON2-3 × HA-SV40-Puro.

For each transfection experiment, about 10^6^ cells PFFs were mixed with 3 μg DNA (1 μg sgRNA-expressing plasmids, and 2 μg Cas9-expressing plasmid) and resuspended in the prewarmed Nucleofector solution. The electroporation was performed with the Nucleofector 2b Device (Lonza), according to the manufacturer’s protocols. The GFP-positive cells were sorted by a FACS Calibur machine (BD Biosciences) and further cultured into clones. Except for PFFs, HEK293T, Huh7, and HepG2 cells were transfected with LentiFit (HANBIO) using the standard protocol when cells reached up to 60% confluence. For example, each 6-well plate cell needs a total of 4 μg plasmids and 6 μL LentiFit reagent. 48 hours after transfection, the cells were collected for RNA and protein extraction.

#### Off-target assay

Three potential off-target sites for each sgRNA were predicted using Cas-OFFinder.^40^ The corresponding PCR products were sequenced. All primers for off-target assay are listed in Table S1.

#### RNA-Seq and analysis

WT and Asgr1^+/–^ pigs were fasted overnight before collection of the liver tissues, *n* = 3 biologically independent repeats per group. All 6 samples were collected and sent to the Beijing Genomics Institute (BGI), where they extracted, quantified, and qualified the total RNA, sequencing 6 samples used BGISEQ platform, averagely generating about 6.79G Gb bases per sample. The average mapping ratio with the reference genome is 90.53%, and the average mapping ratio with the gene is 69.92%; 17109 genes were identified. After obtaining the raw sequencing data, SOAPnuke (v1.5.2) was used for analyzing raw RNA-seq data and trimming the sequencing adapter. HISAT (v2.0.4) was used to align the clean reads to the reference genome and Bowtie2 (v2.2.5) to align the clean reads to the reference genes. The R package DESeq2 was used for differential gene expression analysis, and the read counts matrix was used as the input file. Genes with adjusted q value ≤ 0.05 and |log2FC| ≥ 1 were considered as differentially expressed genes. GO enrichment analysis for differentially expressed genes in a group was carried out using R package phyper. GO terms with a q value ≤ 0.05 were considered significantly enriched. RNA sequencing data are deposited on the NCBI Gene Expression Omnibus (GEO) repository under the accession number GSE268910.

#### Affinity purification by immunoprecipitation (IP)

HepG2 cells stably overexpressing ASGR1 were lysed in IP Lysis buffer (Huaxingbo) on ice for 30 min. Cell debris was removed by centrifugation at 20,000g and 4°C for 10 min. Before IP, 3 mg of cell lysate were precleared with 10 μL Protein A/G-Agarose beads (50% slurry) at 4°C for 2−3 h on a rotator. Protein A/G-Agarose beads were removed by centrifugation at 3000g and 4°C for 5 min. The supernatant (cell lysate) was transferred to a fresh centrifuge tube on ice. Thereafter, the sample was incubated with mouse monoclonal anti-flag antibody (Sigma) or isotype-controlled mouse IgG (Beyotime) overnight at 4°C on a rotary device. Protein A/G-Agarose beads (30 μL) were added and incubated at 4°C for 3 h. The immunoprecipitants (beads) were collected by centrifugation at 5000g and 4°C for 5 min and washed five times with 600 μL IP Lysis buffer. Finally, the immunoprecipitated proteins were eluted by using 2 × loading buffer and subjected to SDS-PAGE, with protein bands visualized by Coomassie blue staining.

#### LC-MS/MS data analysis and protein identification

The IP Solution and Beads samples of HepG2 cells were further analyzed by the China Agricultural University Functional Genomics Platform Biomass Spectrometry Laboratory. “Mascot score” provides an acceptance threshold with false identification probability at a confidence level of 0.05. To reduce the likelihood of false peptide identification, only peptides with Mascot scores ≥ 31 were counted as identified, and each confident protein identification involves at least two unique peptides.

#### Co-immunoprecipitation (Co-IP) for western blot analysis

Half of the 6-well plates cells were transfected to valid the interactions between the candidate proteins and ASGR1. Each well was transfected 4μg DNA (2 μg pEF1a-ASGR1-3 × Flag-IRES-GFP, and 2 μg pEF1a-Candidates-3 × HA-SV40-Puro). 48 hours after transfection, whole-cell extracts were collected by lysis of 1 × 10^7^ cells in 1 mL IP Lysis Buffer (Huaxingbo) with the addition of phenylmethanesulfonylfluoride fluoride (PMSF) protease inhibitor (Beyotime) for 30 min on ice and clarified via centrifugation. For Co-IP, about 400 μL protein extracts were incubated for 12 h at 4°C with anti-HA antibody (Cell Signaling Technology) or control rabbit immunoglobulin G (IgG) (Beyotime), and precipitated proteins were captured by using 30 μL Pierce Protein A/G Agarose (Thermo Fisher Scientific). After five washes with IP Lysis Buffer, bound proteins were eluted in 2 × loading buffer and examined by western blot analysis. Anti-HA antibody (Cell Signaling Technology, 1:1000 dilution) and anti-flag antibody (sigma, 1:1000 dilution) were used for western blot analyses.

#### Hydrodynamic tail vein injection

4-week CD-1 (ICR) male mice from Vital River were selected for hydrodynamic tail vein injection. Rapidly injecting a large volume of DNA solution (∼10% of body weight) via the mouse tail vein can achieve efficient gene transfer and expression *in vivo*, preferentially in the liver.^41^ We followed a previously described injection protocol.^42^ The mice in the experimental group were injected with pEF1a-ASGR1-3 × Flag-IRES-GFP, pEF1a-PON2-3 × HA-SV40-Puro, and pCAG-PBase, at 8 μg each in saline at a volume of 10% body weight. Control groups were injected with pEF1a-ASGR1-3 × Flag-IRES-GFP, pEF1a-3 × HA-SV40-Puro, and pCAG-PBase. Mice were examined on day 4 postinjection.

#### RNA extraction and Real-time quantitative PCR

Following the manufacturer’s protocol, total RNA was isolated from liver tissues or cells (Megan). 2 μg RNA was reverse transcribed using ABScript III RT Master for qPCR (ABclonal). Real-time PCR was performed on LightCycler 480 (Roche). The experimental procedure was carried out according to the kit’s instructions. The relative mRNA expression was analyzed by normalizing with 18S in all genes. The cycle threshold (2^−ΔΔCt^) method was used to calculate the relative expression levels of the genes: ΔCt = Ct target gene − Ct internal reference gene, and ΔΔCt = ΔCt experimental group − ΔCt control group. The sequences of qPCR primers were summarized in Table S1.

#### Immunofluorescence staining

Huh7 cells stably expressing PON2-HA and ASGR1-Flag were fixed with 4% paraformaldehyde for 10 minutes at room temperature after being washed with PBS twice. Then cells were blocked with an Immunol Staining Blocking Buffer (Beyotime) for 30 minutes at room temperature. After that, cells were incubated with primary antibody Flag (Sigma, 1:100 dilution) and HA (Cell Signaling Technology,1:100 dilution) incubated at 4°C overnight. After 5 washes with PBS, cells were incubated with Alexa Fluor 488 goat anti-Mouse IgG (H+L) and Alexa Fluor 594 goat anti-Rabbit IgG (H+L) secondary antibody (Thermo Fisher Scientific, 1:500 dilution) for 1 hour at room temperature with gentle shaking. At last, the samples were washed with PBS 3 times before staining the nucleus with DAPI (Beyotime) for 5 minutes. Immunofluorescence images were obtained and analyzed with Leica Stellaris 5.

#### Immunoblotting

For immunoblotting, whole-cell extracts or liver tissues were homogenized with RIPA buffer and phenylmethanesulfonylfluoride fluoride (PMSF) protease inhibitor (Beyotime) on ice for 30 min, and then centrifugation at 20,000g for 10 min at 4°C, the supernatant was collected. After denaturation, proteins were separated by SDS-PAGE, and then transferred onto a PVDF membrane. The membrane was then blocked with 5% milk for 3 h incubated with appropriate primary antibodies at 4°C overnight, the primary antibodies including β-actin antibody (Beyotime, 1:1000), ASGR1 antibody (Proteintech, 1:2000), PCSK9 antibody (Beyotime, 1:1000), HMGCR antibody (ABclonal, 1:1000), FASN antibody (ABclonal, 1:1000), PON2 antibody (Abcam, 1:20000). After incubation with appropriate secondary antibody, HRP-labeled Goat Anti-Rabbit IgG (H+L) (1:1000) and HRP-labeled Goat Anti-Mouse IgG (H+L) (1:1000) were from Beyotime Institute of Biotechnology, the protein bands were visualized with a SuperSignal West Pico PLUS Kit according to the manufacturer’s instructions (Thermo Fisher Scientific). The images were captured on the Tanon 5200 Imaging System.

#### H&E staining

The liver tissues obtained from WT and ASGR1^+/-^ pigs were fixed in 4% paraformaldehyde for 2 days. The fixed tissues were embedded in paraffin wax and cross-sectioned at 5 μm using a vibratome for H&E staining. The slides were deparaffinized with xylene and subsequently rehydrated with 100%, 90%, 80%, 70%, and 50% alcohol, followed by H_2_O. Finally, the rehydrated slides were stained with a Hematoxylin-Eosin Stain Kit (Solarbio) according to the manufacturer’s instructions and viewed under a fluorescence inversion microscope.

#### Cholesterol and biochemical analysis of serum samples

Before whole blood collection, mice and pigs were fasted overnight. Then pigs' whole blood was collected at the time of euthanasia, while mice’s whole blood was collected from the eyeballs. After standing at room temperature for 1 h, the whole blood was centrifuged at 2000g for 20 min. Then the serum was transferred into new tubes and analyzed in the China Agricultural University Veterinary Teaching Hospital.

### Quantification and statistical analysis

All animal experiments and *in vitro* assays were repeated in at least two independent experiments and all attempts at replication were successful. Statistical analyses were performed with GraphPad Prism software v.9 using an unpaired, two-tailed Student’s t-test when two groups were compared. All data are shown as the mean ± SEM. A *P* value < 0.05 was considered statistically significant.
