# Supplementary material for: ASGR1 is a promising target for lipid reduction in pigs with PON2 as its inhibitor
Source: iScience. 2024 Jun 17;27(7):110288. doi: 10.1016/j.isci.2024.110288 (PMC11269292; doi:10.1016/j.isci.2024.110288)
Supplement: Document S1. Figures S1–S4 and Tables S1 and S2 [file mmc1.pdf]

iScience, Volume *27*

Supplemental information

ASGR1 is a promising target for lipid reduction in pigs with PON2 as its inhibitor

Yunjun Yin, Jun Liu, Jia Yu, Dingcai Dong, Fei Gao, Libao Yu, Xuguang Du, and Sen Wu

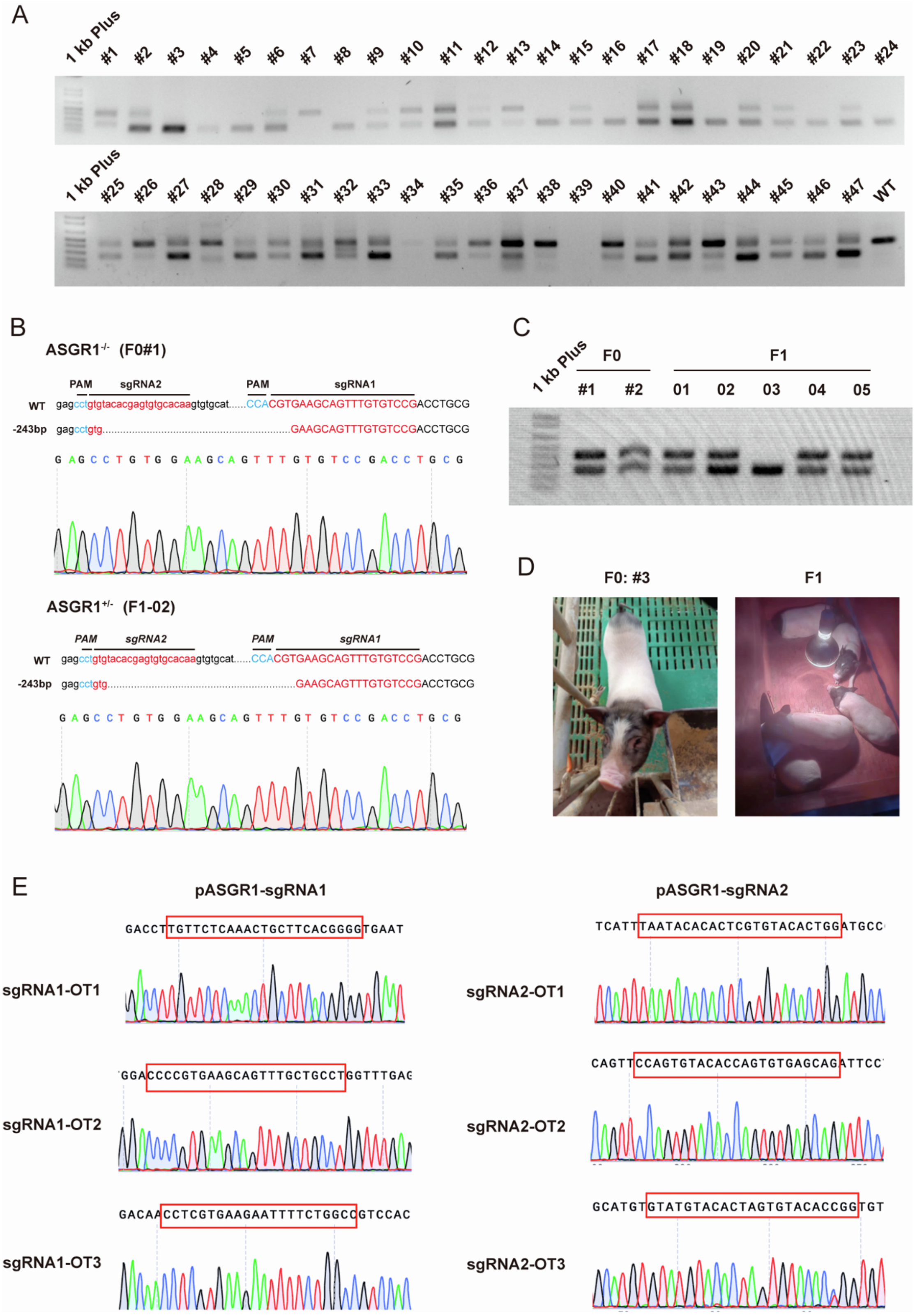

Figure S1. Generation and genotyping of ASGR1 Knockout pigs, related to Figure 1.

(A) PCR genotyping of porcine fetal fibroblast clones at the ASGR1 loci. (B) Representative sequencing chromatograms of ASGR1$^{-/-}$ (F0#1) and ASGR1$^{+/-}$ (F1-02) pigs. (C) PCR genotyping of pigs' gender. (D) Representative photograph of F0 and F1 pigs. (E) Analysis of the three potential off-target sites for each sgRNA in ASGR1$^{+/-}$ pigs.

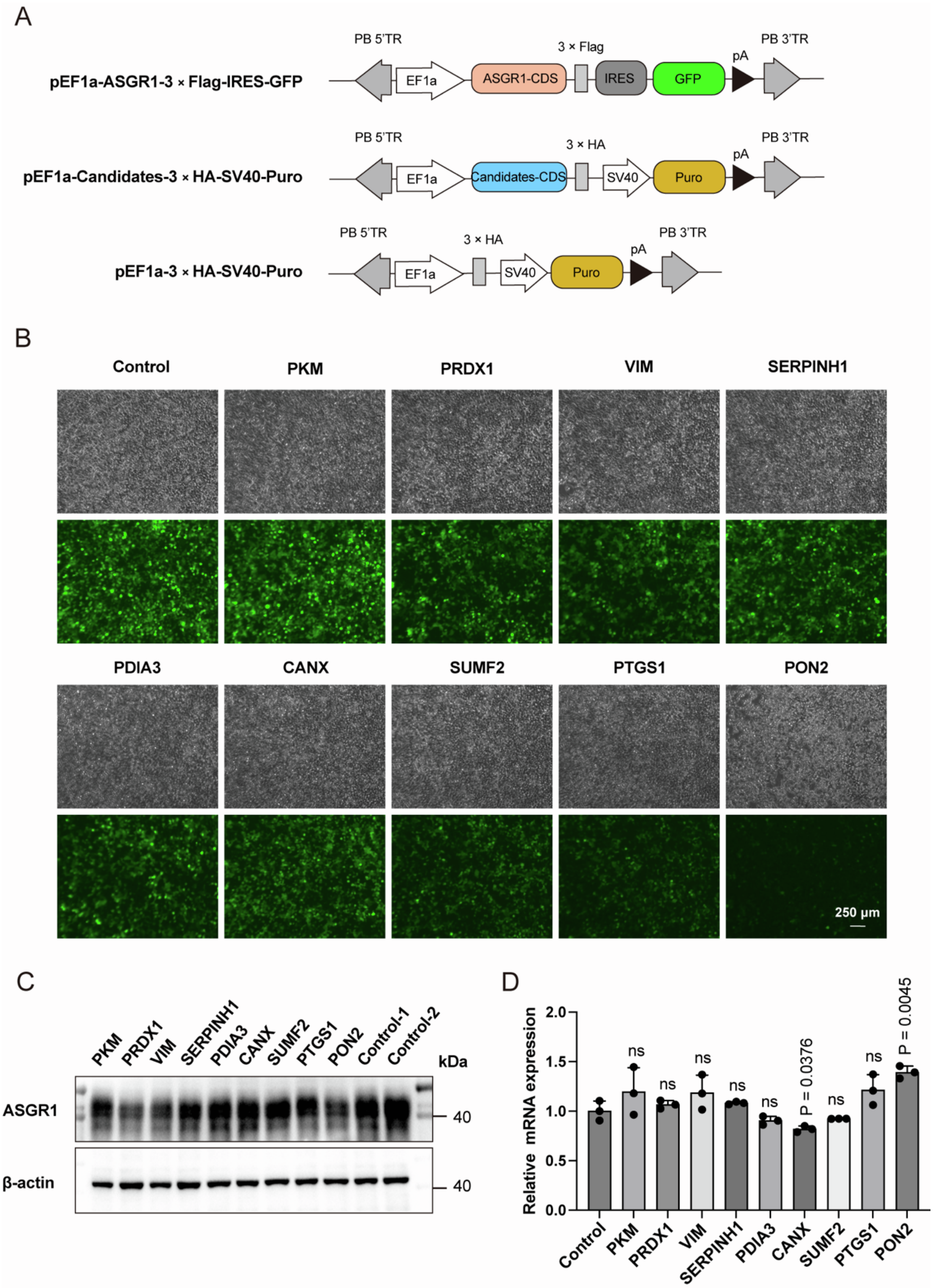

Figure S2. Validation of the selected ASGR1-interacting candidate proteins, related to Figure 4.

(A) Schematic of the ASGR1 and candidate proteins overexpression vector. 3 × Flag or HA was fused in the C terminal of genes. IRES, internal ribosome entry site; pA, polyadenylation signal. (B) Equal amounts of pEF1a-ASGR1-3 × Flag-IRES-GFP and pEF1a-Canditates-3 × HA-SV40-Puro plasmids were co-expressed transiently in HEK293T cells, and the control group was transfected with pEF1a-ASGR1-3 × Flag-IRES-GFP and pEF1a-3 × HA-SV40-Puro empty vector. The expression of green fluorescence in cells can reflect the expression of ASGR1. (Scale bars, 250 μm). (C) Immunoblotting analysis of the protein level of ASGR1 in the HepG2 cells. (D) Expression of ASGR1 in HepG2 cells measured by RT–qPCR (n = 3). All values are presented as mean ± SEM. P values were calculated by unpaired two-tailed Student's t-test.

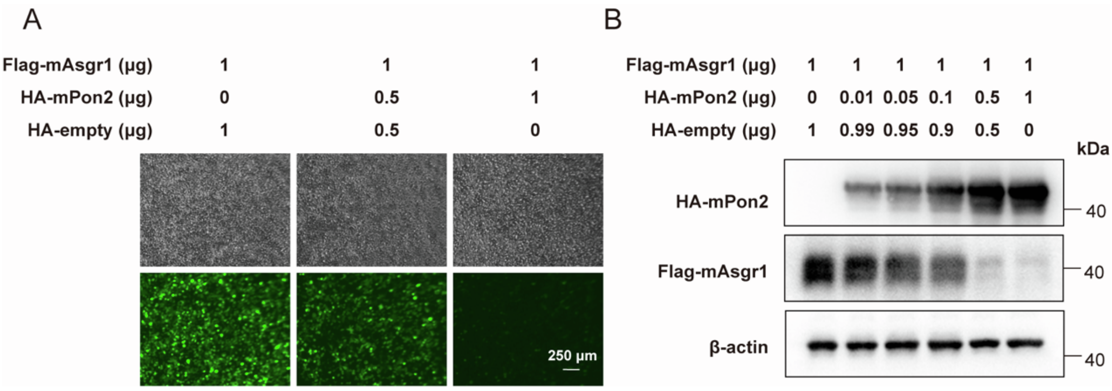

Figure S3. mPon2 downregulates mAsgr1 protein level in a dose-dependent manner, related to Figure 5. (A) HEK293T cells were transfected with mAsgr1 and different doses of mPon2 (Scale bars, 250 μm), and additional pEF1a-3 × HA-SV40-Puro empty vector was transfected to ensure consistency in the total amount of plasmid DNA introduced into the cells. (B) Immunoblotting analysis of the protein levels of mPon2 and mAsgr1.

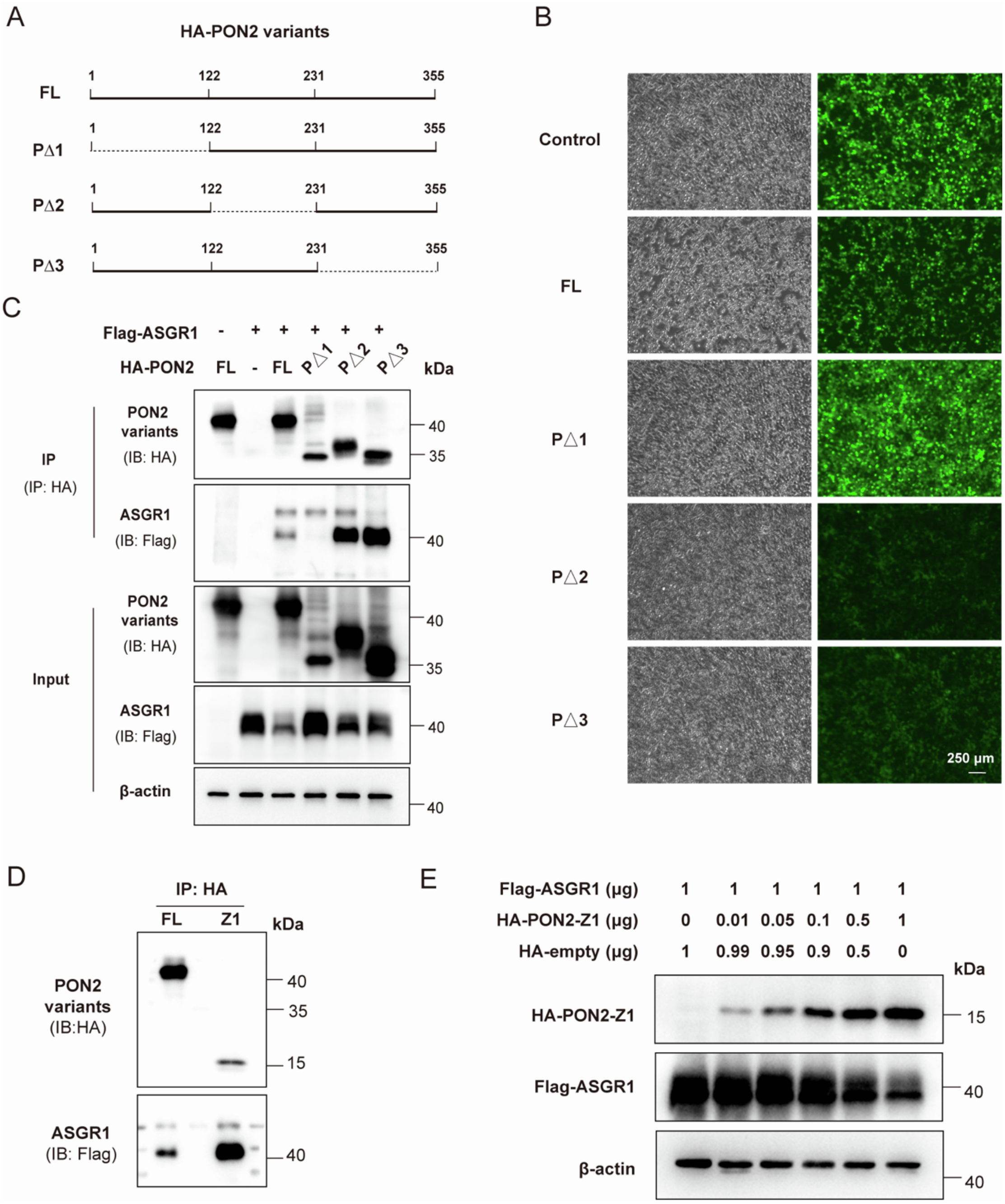

Figure S4. Identification of the region (a.a. 1-122) of PON2 as the ASGR1-interacting Sequence. (A) Schematic of various PON2 truncations. (B) Co-expression of the PON2-HA variants and ASGR1-Flag in HEK293T cells (Scale bars, 250 μm). (C) Co-IP of the PON2-HA variants and ASGR1 in HEK293T cells. (D) Co-IP of the PON2-HA variants and ASGR1 in HEK293T cells. Pull-downs with HA antibodies were analyzed by Immunoblotting for Flag (ASGR1) and HA (PON2-HA variants). Isotype-controlled rabbit "IgG" as a negative

control. “Input” represents 10% of the original material subjected to coimmunoprecipitation.

(E) Immunoblotting analysis of the expression of PON2-Z1 and ASGR1.

Table S1. Primers sequences, related to STAR Methods.

	Primers sequences	
Primers name	Forward (5'-3')	Reverse (5'-3')
pASGR1-sgRNA1	TTGTGCACACTCGTGTACAC	
pASGR1-sgRNA2	CGGACACAAACTGCTTCACG	
sgRNA1-OT1	TGTTCTCAAACTGCTTCACG	
sgRNA1-OT2	AGGCAGCAAACTGCTTCACG	
sgRNA1-OT3	CGGCCAGAAAATTCTTCACG	
sgRNA2-OT1	TAATACACACTCGTGTACAC	
sgRNA2-OT2	CTGCTCACACTGGTGTACAC	
sgRNA2-OT3	GTATGTACACTAGTGTACAC	
sgRNA1-OT1-PCR	CTGTGTTCTTAAGGTTTGCTTGG	CTTTCAGAATTAAAGCCCCTTGT
sgRNA1-OT2-PCR	TTTGATCTGTGCTGTGTATCCTG	GAGGAGAAAAGGGTGTGTGTATG
sgRNA1-OT3-PCR	TTCTTCTTTCCTTGTAGGGAACC	GAATTCAAACTTGTTCAGGATCG
sgRNA2-OT1-PCR	GTGATTAAGGCAAGAAGCCCTAT	GGGCTACTGTATTGTGAGTGGTC
sgRNA2-OT2-PCR	GTGAGCTGAGTGGCTGCTCTGC	AACTGAGCAGAGTCTCAGATTGG
sgRNA2-OT3-PCR	GAGTGCTTCTGGCTAAACAAAAA	AGCATCTTCACTTTCAGCATCTC
pASGR1-KO-PCR	AATGTGGGCAGAAAGATGAAGT	GGACAGAGGGAAGGTGAGG
18S-qPCR	CCTGCGGCTTAATTTGACTC	ATGCCAGAGTCTCGTTCGTT
GAPDH-qPCR	GTGGACCTGACCTGCCGTCT	GGAGGAGTGGGTGTCGCTGT
hASGR1-qPCR	GAGAGAGACGTTCAGCAACTTC	GGGACTCTAGCGACTTCATCTT
pIL-6-qPCR	GGACGCCTGGAAGAAGATGC	TGCCAGTACCTCCTTGCTGTT
pTNF-α-qPCR	ACCACGCTCTTCTGCCTACTG	CTCGGCTTTGACATTGGCTACA
pNLRP3-qPCR	CTACCCTGGCAGGCATCATT	CTTGTTCCGAGCCCAGTTCT
pNF-kB-qPCR	GAGGCCGTAAATCTCTCTGGG	AGCCAGCAGCATCTTCACAT
mIL1β-qPCR	TGGACCTTCCAGGATGAGGACA	GTTCATCTCGGAGCCTGTAGTG
mTNF-α-qPCR	GGTGCCTATGTCTCAGCCTCTT	GCCATAGAACTGATGAGAGGGAG
mHmgcr-qPCR	GCTCGTCTACAGAAACTCCACG	GCTTCAGCAGTGCTTTCTCCGT
mPcsk9-qPCR	ATGGCACCAGACAGAGGAAGAC	CACGCTGTTGAAGTCGGTGATG
mLdlr-qPCR	GAATCTACTGGTCCGACCTGTC	CTGTCCAGTAGATGTTGCGGTG
mSrebp1-qPCR	CGACTACATCCGCTTCTTGCAG	CCTCCATAGACACATCTGTGCC
mScd1-qPCR	GCAAGCTCTACACCTGCCTCTT	CGTGCCTTGTAAGTTCTGTGGC
mSrebp2-qPCR	AGAAAGAGCGGTGGAGTCCTTG	GAACTGCTGGAGAATGGTGAGG
mFasn-qPCR	GCTGCGGAAACTTCAGGAAAT	AGAGACGTGTCACTCCTGGACTT

Table S2. List of 23 ASGR1-interacting proteins that appear in both Solution and Beads samples, related to Figure 3.

UniProt ID	Protein name	Description	Score		Coverage (%)		PSM	
Samples			Solution	Beads	Solution	Beads	Solution	Beads
P08729	KRT7	Keratin, type II cytoskeletal 7	2314	2795	54	45	71	82
P05783	KRT18	Keratin, type I cytoskeletal 18	610	1554	35	69	23	62
P08727	KRT19	Keratin, type I cytoskeletal 19	818	593	58	36	35	25
Q13751	LAMB3	Laminin subunit beta-3	661	1498	19	43	17	51
P50454	SERPINH1	Serpin H1	1046	1163	54	50	30	32
P08670	VIM	Vimentin	1597	825	69	53	49	24
P27824	CANX	Calnexin	1466	753	53	25	52	23
Q8TEM1	NUP210	Nuclear pore membrane glycoprotein 210	153	414	4	11	6	21
P14618	PKM	Pyruvate kinase	323	374	30	35	12	15
Q12797	ASPH	Aspartyl/asparaginyl beta-hydroxylase	281	369	16	21	8	20
Q9Y446	PKP3	Plakophilin-3	110	311	9	18	5	12
H0Y612	TRIM33	E3 ubiquitin-protein ligase TRIM33	69	302	4	15	3	12
Q9ULG6	CCPG1	Cell cycle progression protein 1	61	250	5	15	5	9
A0A0J9YXF2	PON2	Paraoxonase 2	242	227	39	33	9	12
A0A0C4DGV8	SEMA3B	Semaphorin-3B	377	210	23	14	15	7
P30101	PDIA3	Protein disulfide-isomerase A3	128	189	21	28	6	11
P42677	RPS27	ribosomal protein S27	60	144	25	39	2	5
J3KQJ1	SUMF2	Sulfatase-modifying factor 2	132	105	16	21	5	5
J3KQE5	RAN	GTP-binding nuclear protein Ran	65	94	11	14	2	3
Q13444	ADAM15	Disintegrin and metalloproteinase domain-containing protein 15	166	72	7	3	4	2

G5E9W2	CHPF2	Chondroitin sulfate glucuronyltransferase	77	70	5	5	4	4
A0A0A0MSI0	PRDX1	Peroxiredoxin-1	146	60	39	17	7	4
P23219	PTGS1	Prostaglandin G/H synthase 1	127	216	10	17	4	10

Abbreviation: PSM, peptide-spectrum match
